# Development of New Stringency Indices for Nonpharmacological Social Distancing Policies Implemented in Korea During the COVID-19 Pandemic: Random Forest Approach

**DOI:** 10.2196/47099

**Published:** 2024-01-08

**Authors:** Catherine Apio, Kyulhee Han, Doeun Lee, Bogyeom Lee, Taesung Park

**Affiliations:** 1 Interdisciplinary Program in Bioinformatics Seoul National University Seoul Republic of Korea; 2 Ross School of Business University of Michigan-Ann Arbor Ann Arbor, MI United States; 3 Department of Statisitcs Seoul National University Seoul Republic of Korea

**Keywords:** COVID-19, restriction policy, Stringency Index, Korea Stringency Index, social distancing, physical distancing, pandemic, government, restriction, effectiveness, policy

## Abstract

**Background:**

In the absence of an effective treatment method or vaccine, the outbreak of the COVID-19 pandemic elicited a wide range of unprecedented restriction policies aimed at mitigating and suppressing the spread of the SARS-CoV-2 virus. These policies and their Stringency Index (SI) of more than 160 countries were systematically recorded in the Oxford COVID-19 Government Response Tracker (OxCGRT) data set. The SI is a summary measure of the overall strictness of these policies. However, the OxCGRT SI may not fully reflect the stringency levels of the restriction policies implemented in Korea. Korea implemented 33 COVID-19 restriction policies targeting 4 areas: public facilities, public events, social gatherings, and religious gatherings.

**Objective:**

This study aims to develop new Korea Stringency Indices (KSIs) that reflect the stringency levels of Korea’s restriction policies better and to determine which government-implemented policies were most effective in managing the COVID-19 pandemic in Korea.

**Methods:**

The random forest method was used to calculate the new KSIs using feature importance values and determine their effectiveness in managing daily COVID-19 confirmed cases. Five analysis periods were considered, including November 01, 2020, to January 20, 2021 (Period 1), January 20, 2021, to June 27, 2021 (Period 2), November 01, 2020, to June 27, 2021 (Period 3), June 27, 2021, to November 01, 2021 (Period 4), and November 01, 2021, to April 24, 2022 (Period 5).

**Results:**

Among the KSIs, public facilities in period 4, public events in period 2, religious gatherings in periods 1 and 3, and social gatherings in period 5 had the highest importance. Among the public facilities, policies associated with operation hour restrictions in cinemas, restaurants, PC rooms, indoor sports facilities, karaoke, coffee shops, night entertainment facilities, and baths or saunas had the highest importance across all analysis periods. Strong positive correlations were observed between daily confirmed cases and public facilities, religious gatherings, and public events in period 1 of the pandemic. From then, weaker and negative correlations were observed in the remaining analysis periods. The comparison with the OxCGRT SI showed that the SI had a relatively lower feature importance and correlation with daily confirmed cases than the proposed KSIs, making KSIs more effective than SI.

**Conclusions:**

Restriction policies targeting public facilities were the most effective among the policies analyzed. In addition, different periods call for the enforcement of different policies given their effectiveness varies during the pandemic.

## Introduction

The spread of COVID-19 [1-3], which became a global pandemic [4], elicited a wide range of unprecedented restriction policies from different governments aimed to mitigate and suppress the spread of the SARS-CoV-2 virus [5-7]. Without a vaccine or an effective treatment, these social distancing policies included school closures, travel restrictions, bans on social and private gatherings, stay-at-home orders, workplace closures, closure of public transportation, and so forth [6]. The transmission of the SARS-CoV-2 virus spreads through contacts made between susceptible and infectious individuals depending on the spatial distance between contacts. Therefore, the suppression of contacts is the goal of these policies, designed to slow the growth rate of infections, as inferred from past epidemics [8,9].

The impact of implemented government policies in lowering COVID-19 cases has been demonstrated in many studies as the pandemic progressed [6,10-15]. One country that implemented social distancing policies was South Korea. When the first patient with COVID-19 was confirmed in Korea on January 20, 2020 [16-18], voluntary social distancing started to be practiced by citizens in their daily lives until it was declared a global pandemic. With the declaration of COVID-19 as a worldwide pandemic on March 11, 2020 [4], the Korean government instituted its first social distancing for 2 weeks starting March 22, 2020, when the number of cases rose to approximately 100-150 daily cases [19]. The government combined testing, contact tracing, early isolation, and the free treatment of positive cases together with digital technologies without considering “lockdown” measures [20-22]. Many countries and governments worldwide applauded South Korea’s response as the most influential and was considered as one of the most effective models against COVID-19 in the early months that followed the pandemic [20]. The COVID-19 curve was flattened to an average of 6.4 daily cases in the first week of May 2020 [20-22]. However, as the pandemic progressed, this approach could not hold up due to worsening COVID-19 situations in the country, mainly in the metropolitan areas tied to small church gatherings, restaurants, social gatherings, nightclubs, and schools [17,18,23,24]. From then on, the government imposed restrictions in these areas and would strengthen or lower the restriction levels depending on the average daily confirmed cases. The policies and their levels would be announced via official government websites and press releases [25].

The policies implemented by different countries worldwide were systematically collected and recorded by the Blavatnik School of Government, and the University of Oxford called the Oxford COVID-19 Government Response Tracker (OxCGRT) data set [26]. This data recorded more than 17 policies and indices from more than 160 countries. This also included the Stringency Index (SI), a summary of the measure of the overall strictness of these policies. Previously, we conducted studies showing the relationship between these policies and indices in lowering COVID-19 cases in more than 90 countries and South Korea [13,27]. However, we observed that the SI provided by the OxCGRT data may not fully capture Korea’s containment and closure policies. To describe government responses simply, OxCGRT would summarize the policies implemented in each country into the same number of levels and then use an additive method to calculate the unweighted SI since the approach is transparent and easiest to interpret. However, this approach cannot capture small policy changes across all countries and adequately reflect the influence of different policies on an individual basis. Some countries applied more stringent policy changes that cannot be captured by the calculated OxCGRT SI. For example, South Korea implemented restriction policies targeting social gatherings and would relax or tighten the policies by increasing or decreasing the maximum number of people allowed at these social gatherings like for 8 to 10 or vice versa. These changes were too subtle to be reflected in the OxCGRT’s SI. Second, OxCGRT did not consider the impact of each policy on the spread of COVID-19 when calculating SI. This assumes that each policy will have an equal influence and contribution to the final policy score. OxCGRT encouraged users to carefully consider which combination and weighting of policies would best capture the dimensions they are seeking to measure [26]. Therefore, Korea Stringency Indices (KSIs) better capture the subtle changes present in Korea’s COVID-19 response policies. In addition, we can focus on the target areas of the policies and tell whether the implemented policies and their level of strictness are having an impact on the people who frequent those target areas.

Although Korea was one of the first countries to be affected by COVID-19 [28], many countries, like China, Uganda, Europe, and the United States, implemented a wide range of stringent restriction policies in response to the rapid increase in the daily number of COVID-19 cases. But, after experiencing a sharp growth in COVID-19 cases early in the pandemic, Korea rapidly controlled transmission while implementing less stringent social distancing measures than other countries. The government only implemented free nationwide public testing, isolation of infected individuals, vaccination, and information of the public through text messages together with government press conferences [21,29]. The government maintained this course of less strict policies even when the daily cases rose to tens of thousands without locking down regions and causing severe economic damage [28]. Analyzing the effectiveness of these policies in Korea can answer questions about which policies were most effective at managing the pandemic situation in Korea and provide a framework for responses that can be adopted against future pandemics without resorting to stringent measures that affect people mentally and the economy. Maybe we can also find loopholes in Korea’s response strategy and improve its efficiency.

In our analysis, we aim to answer the question of which implemented government policies were most effective in managing the COVID-19 situation in Korea and calculate KSI using the COVID-19 restriction policies implemented in Korea. The Korean government implemented restriction policies targeting 4 target areas: public facilities, public events, religious gatherings, and social gatherings. The policies and their level of strictness were manually obtained from this website [25]. Our final goal is to provide KSI, which is the summarized score to express the strictness of the policies considering their impact on the spread of COVID-19. We first assumed that each policy may have different impacts on the spread of COVID-19. Given this reason, the impact of these policies on the spread of COVID-19 was estimated using a random forest (RF) model. In addition, RF is not restricted by the multiple levels that are present in a given policy. All the information given by the levels in each given policy is used to estimate the impact (feature importance) of the policy on the spread of COVID-19. These feature importance values are taken as the weights of the policies and used for calculating the summarized score of each policy category. The basis for this assumption is that even if the amount of change in 2 policies is the same, the more important policy should be weighted more in the summarized score. Finally, we assumed that the impact of each policy on COVID-19 could be different by each period. Therefore, the analysis was carried out in 5 segments or periods corresponding to waves of the pandemic or turning points during the pandemic. Such turning points classify the behavior of a country’s trajectory throughout the pandemic as being in (or over) their subsequent waves.

## Methods

### Data

The COVID-19 daily series of confirmed cases in Korea was downloaded from the “Our World in Data” (OWID) website [30]. Countries implemented different restriction policies with the progress of the pandemic, and these policies were recorded by the OxCGRT [26,31]. The OxCGRT data systematically recorded the different levels of the policies on an ordinal or numeric scale. The SI was obtained from the OxCGRT data and records the average level of strictness of the policies that primarily contain and restrict people’s behavior and movements (a summary of the measure of the overall strictness of these policies).

### Korea’s Restriction Policies

The COVID-19 restriction policies implemented in Korea and their strictness were collected manually from the government’s official website of the Korea Disease Control and Prevention Agency (KDCA) in response to COVID-19 [25]. The KDCA gave press releases about the COVID-19 situation in the country. The social distancing policies and their strictness levels for a given period were announced during these press releases and also on the official KDCA website. All press release posts by the KDCA for our analysis period (November 01, 2020, to April 24, 2022) were checked (1532 posts), and the social distancing policy level for the announced given period was recorded in a spreadsheet with their corresponding dates. If there were changes to the social distancing policy, they were summarized in the spreadsheet for the given date. However, if there was no updated policy notice for a certain period, we assumed the same policy level had been maintained. These policies focused on 4 main activities with different targets under each, including public facilities, religious gatherings, public events, and social gatherings, as summarized in Table 1. The level of strictness or stringency of the policies varied with the number of confirmed cases and is outlined in the “score” column of Table 2. A higher score value means a more vital constraint.

**Table 1:** List of activities and target places focused on by Korea’s restriction policies.

Category and target			Policy
**Public facilities**			
	**Night entertainment facilities**		
		Operating hours restrictions	Policy 1
		Prohibition of indoor eating	Policy 2
	**Restaurant**		
		Operating hours restrictions	Policy 3
		Prohibition of indoor eating	Policy 4
	**Coffee shop**		
		Operating hours restrictions	Policy 5
		Prohibition of indoor eating	Policy 6
	**Karaoke**		
		Operating hours restrictions	Policy 7
		Prohibition of indoor eating	Policy 8
	**Baths or saunas**		
		Operating hours restrictions	Policy 9
		Prohibition of indoor eating	Policy 10
	**Indoor sports facilities**		
		Operating hours restrictions	Policy 11
		Prohibition of indoor eating	Policy 12
	**Sales promotion center**		
		Operating hours restrictions	Policy 13
		Prohibition of indoor eating	Policy 14
	**Cinema**		
		Operating hours restrictions	Policy 15
		Prohibition of indoor eating	Policy 16
	**Supermarket**		
		Operating hours restrictions	Policy 17
		Prohibition of indoor eating	Policy 18
	**PC rooms**		
		Operating hours restrictions	Policy 19
		Prohibition of indoor eating	Policy 20
**Public events**			
	**Personnel**		
		Number of gathering restrictions (all people)	Policy 21
		Number of gathering restrictions (vaccinated people)	Policy 22
**Religious gatherings**			
	**Personnel**		
		Number of gathering restrictions (all people)	Policy 23
		Number of gathering restrictions (vaccinated people)	Policy 24
**Social gathering**			
	**Personnel**		
		Number of gathering restrictions (all people)	Policy 25
		Number of gathering restrictions (unvaccinated people)	Policy 26
		Number of gathering restrictions (penalty after 6 PM)	Policy 27
	**Exceptions**		
		Number of gathering restrictions (family living together, families needing care, and those on their deathbed)	Policy 28
		Number of gathering restrictions (sports)	Policy 29
		Number of gathering restrictions (immediate family)	Policy 30
	**Restaurants and coffee shops**		
		Number of gathering restrictions (vaccinated people)	Policy 31
		Number of gathering restrictions (unvaccinated people)	Policy 32
		Number of gathering restrictions (all people)	Policy 33

**Table 2:** A summary of Korea’s level of strictness for each restriction policy.

Category, policy, and explanation			Score
**Public facilities**			
	**Operating hours restrictions**		
		Prohibition of operating	10
		Allowing operation until 9:00 PM	7
		Allowing operation until 10:00 PM	5
		Allowing operation until 11:00 PM	4
		Allowing operation until 12:00 PM	3
		No restrictions	1
	**Prohibition of indoor eating**		
		Prohibition of operating	10
		Prohibition of indoor eating	5
		No restrictions	1
**Public events**			
	**Number of gathering restrictions—for all people**		
		Prohibition of gathering	10
		Limited to 49	6
		Limited to 99	5
		Limited to 250	4
		Limited to 299	3
	**Number of gathering restrictions—for vaccinated group**		
		Prohibition of gathering	10
		Limited to 49	6
		Limited to 99	5
		Limited to 250	4
		Limited to 299	3
		Limited to 499	2
		No restrictions	1
**Religious gatherings**			
	**Number of gathering restrictions—for all people**		
		Prohibition of gathering	10
		Limited to 10% of maximum occupancy	6
		Limited to 20% of maximum occupancy	5
		Limited to 30% of maximum occupancy	4
		Limited to 50% of maximum occupancy	3
		Limited to 70% of maximum occupancy	2
		No restrictions	1
	**Number of gathering restrictions—for vaccinated group**		
		Prohibition of gathering	10
		Limited to 10% of maximum occupancy	6
		Limited to 20% of maximum occupancy	5
		Limited to 30% of maximum occupancy	4
		Limited to 70% of maximum occupancy	2
		No restrictions	1
**Social gatherings**			
	**Number of gathering restrictions—for all people**		
		Limited to 4	6
		Limited to 6	5
		Limited to 8	4
		Limited to 10	3
		No restrictions	1
	**Number of gathering restrictions—for nonvaccinated group**		
		Limited to 2	7
		Limited to 4	6
		Limited to 6	5
		Limited to 7	4
		Limited to 10	3
		No restrictions	1
	**Number of gathering restrictions—a penalty after 6 PM**		
		Penalty on the number of gatherings after 6:00 PM	2
		No penalty	1
	**Exceptions: family living together, a family who needs care, a deathbed**		
		Same restrictions on these people (No exception)	2
		No restrictions on these people	1
	**Exceptions: sports**		
		Same restrictions on these people (No exception)	2
		No restrictions on these people	1
	**Exceptions: immediate family**		
		Same restrictions on these people (No exception)	2
		No restrictions on these people	1
	**Restaurant and coffee shop: vaccinated people**		
		Limited to 4	6
		Limited to 6	5
		Limited to 8	4
		Limited to 10	3
		No restrictions	1
	**Restaurant and coffee shop: nonvaccinated people**		
		Limited to 1	8
		Limited to 2	7
		Limited to 4	6
		Limited to 6	5
		Limited to 7	4
		Limited to 10	3
		No restrictions	1
	**Restaurant and coffee shop: sharing a table with nonvaccinated people**		
		No table sharing between nonvaccinated and vaccinated persons	2
		No restrictions	1

### Analysis Periods

**Figure 1:**
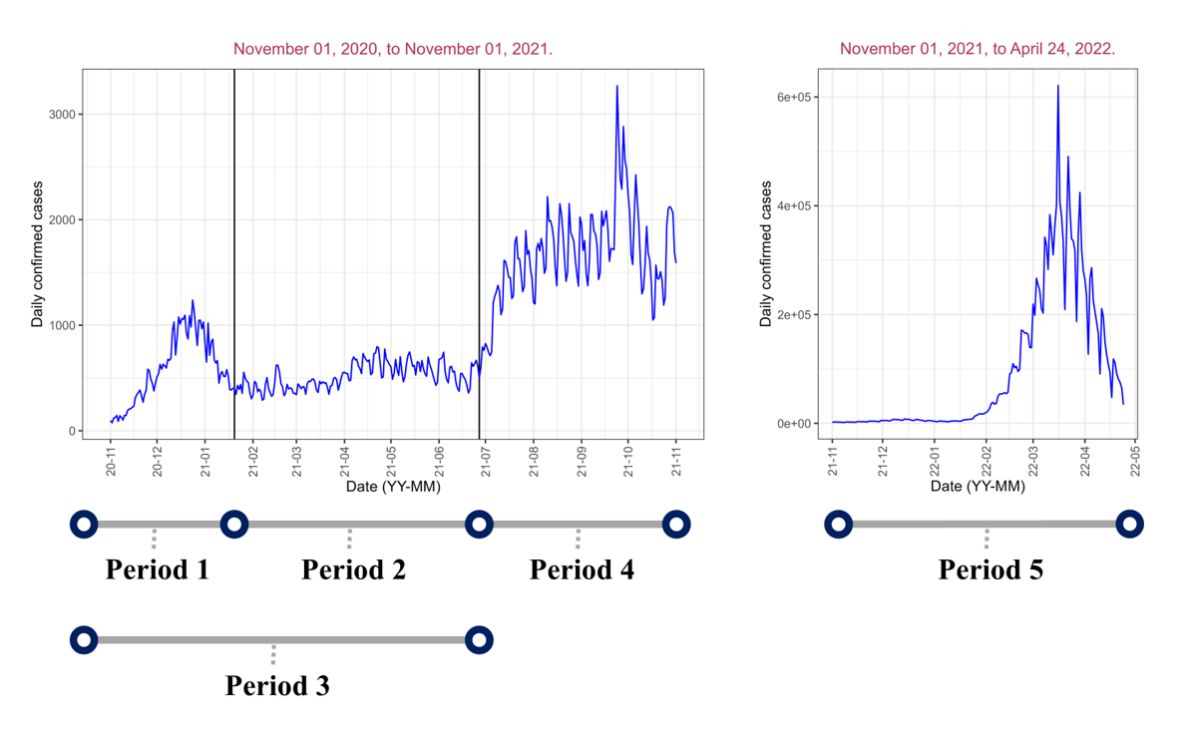
The 5 analysis periods used in the study.

The analysis was carried out in 5 periods. Period 1 corresponds to the third wave of the pandemic ranging from November 01, 2020, to January 20, 2021. Period 2 comes after the 3rd wave of the pandemic and ranges from January 20, 2021 to June 27, 2021. Period 3 combines the first and second analysis periods (November 01, 2020, to June 27, 2021). Period 4 consists of the summer vacation period and right after the summer vacation period, from June 27, 2021, to November 01, 2021. Period 5 is from November 01, 2021, to April 24, 2022, the step-by-step recovery period. This period is characterized by the major cities in the country taking steps to go back to normal operations like before the pandemic and the end of the social distancing policies except masking. The analysis period is summarized in Figure 1 below. The end of the analysis period 4 is the beginning of period 5 but with a different scale for the y-axis.

### Statistical Analysis

In the first step, an RF model was used to estimate the feature importance of policies corresponding to each target level policy, respectively against COVID-19 daily confirmed cases. Since there are 15 target policies, 15 RF models for each target policy were fitted each independent of the other target policies. Then, the estimated feature importance of the 33 policies was multiplied by their respective policy “score” values and summed to form the 15 policies according to the target category level as shown in Table 1. Public facilities had 10 target policies, public events and religious gatherings had 1 target policy each (personnel), and social gatherings had 3 target policies (restaurants and cafes, exceptions, and personnel). In the second step, another RF model was fitted with the 15 target-level policies and their feature importance was estimated. Then, the KSI for each category is estimated by multiplying the estimated feature importance of each target policy with the calculated target policies estimated above and summed corresponding to each category. The RF model is an ensemble learner based on randomized decision trees and provides different feature importance measures, one from statistical permutation tests and the other from training an RF classifier. Both measures have been found to correlate reasonably well and provide excellent means of measuring feature relevance [32]. Therefore, although simple, the calculation of KSIs using this method is justifiable. Since there are 4 categories, we will get 4 KSI values, 1 for each category. In the last step, an RF model is used to rank the impact of the restriction policies using KSIs on COVID-19 daily cases using feature importance. The SI from the OxCGRT data was included in the analysis.

During the analysis, a training validation approach was used to find optimal hyperparameters, with the last 14 days used as a validation set in each analysis period. A detailed explanation of hyperparameter settings is explained in Table 3 below.

**Table 3:** Optimal hyperparameter settings used in the analysis.

Hyperparameter	Range	Explanation
# of trees	[4, 8, 16, 32, 64, 100]	The number of trees in the forest.
Max features	[‘auto’, ‘sqrt’]	The number of features to consider at every split. Auto: max features = # of features. Sqrt: max features = sqrt (# of features)
Max depth	[None, 10, …, 110]	The maximum number of levels in the tree. None: no limit to the depth of the tree.
Min sample split	[2, 5, 10]	The minimum number of samples required to split a node.
Min sample leaf	[1, 2, 4]	The minimum number of samples required at each leaf node.

### Ethical Considerations

No individual data were used in our study. Only cumulative count data of anonymous individuals were used. Since publicly available data were used for this study, ethical approval from a research board was unnecessary.

## Results

### Feature Importance of the Policies

**Figure 2:**
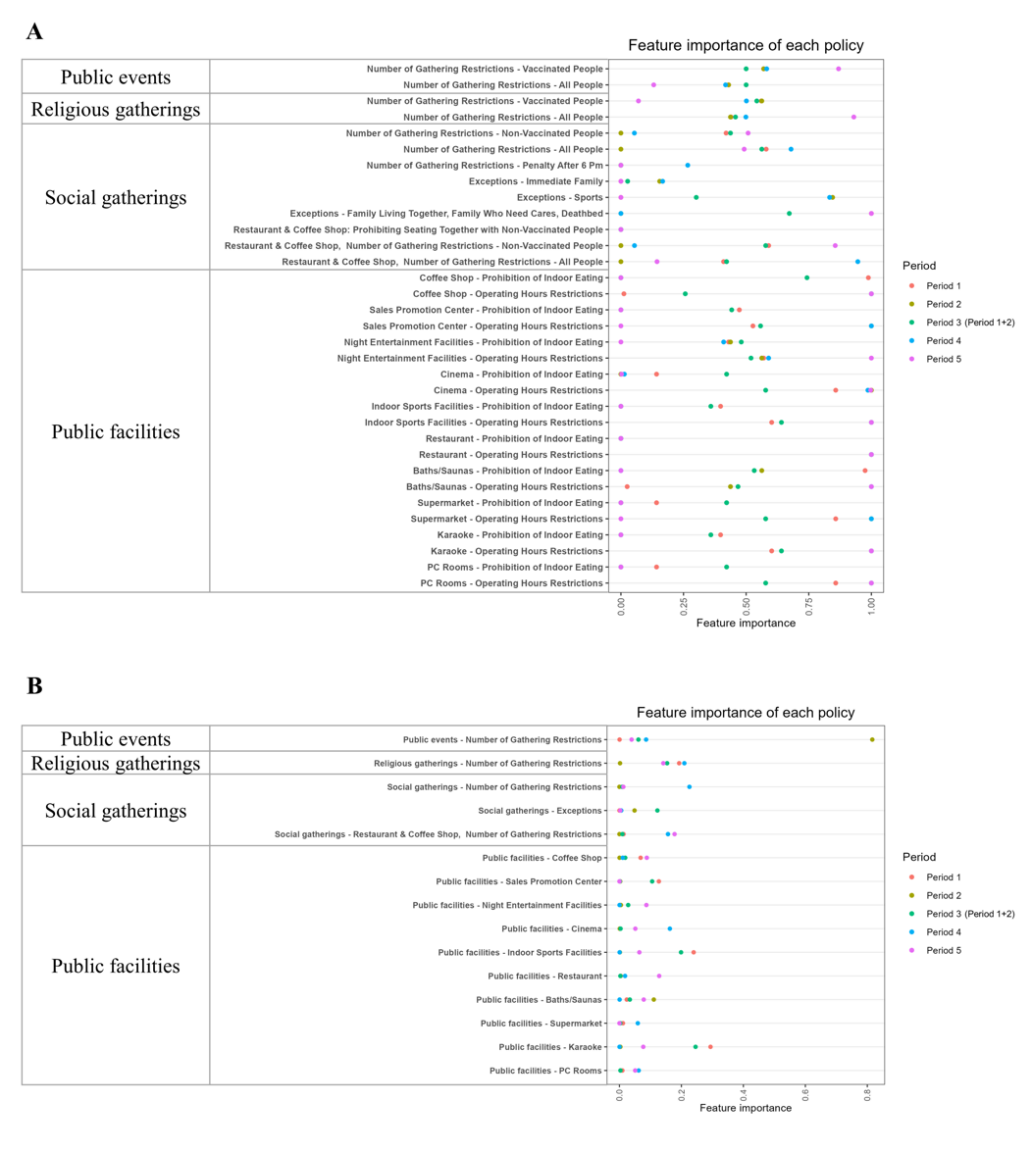
The feature importance values were obtained from RF models across the 5 analysis periods. (A) Results from the 15 RF models for the 33 policies for each period. (B) Results from 1 RF model for the 15 target policies for each period. RF: random forest.

The feature importance of the 33 policies and the 15 target policies for the 5 analysis periods is summarized in Figure 2. The feature importance of the 33 policies varies much across the analysis periods and we observe a pattern of low, intermediate, and high feature importance among the policies and across the 5 analysis periods. Most restriction policies targeting public events and religious gatherings had their feature importance around 0.5 across the analysis periods except for period 5. Restriction policies targeting public facilities also had their importance mainly around 0.50, except in period 5 which had importance values of either 0 or 1.00. The most prominent impact was observed with the restriction policies that targeted the use of public facilities. A few policies stood out, especially in the step-by-step recovery period (period 5), including operating hour restrictions on cinemas, restaurants, PC rooms, indoor sports facilities, karaoke, coffee shops, night entertainment facilities, and baths or saunas. After the summer vacation (period 4), operating hour restrictions were imposed on supermarkets, cinemas, sales promotion centers, restaurants and coffee shops. Period 3 which combines periods 1 and 2 had feature importance for most policies at around 0.50. We conclude that most policies associated with operating hour restrictions followed by the number of gathering restrictions had higher feature importance values than the other policies for a given analysis period, respectively.

There was not much variation in the feature importance of the 15 target policies (Figure 2B) obtained from summing up the 33 policies across the 5 analysis periods. All feature importance was below 0.40, except for the restriction on the number of gatherings in public places in period 2 (0.80). For some policies, their importance was either equal to or near 0 at a given analysis period.

### Korea Stringency Indices

**Figure 3:**
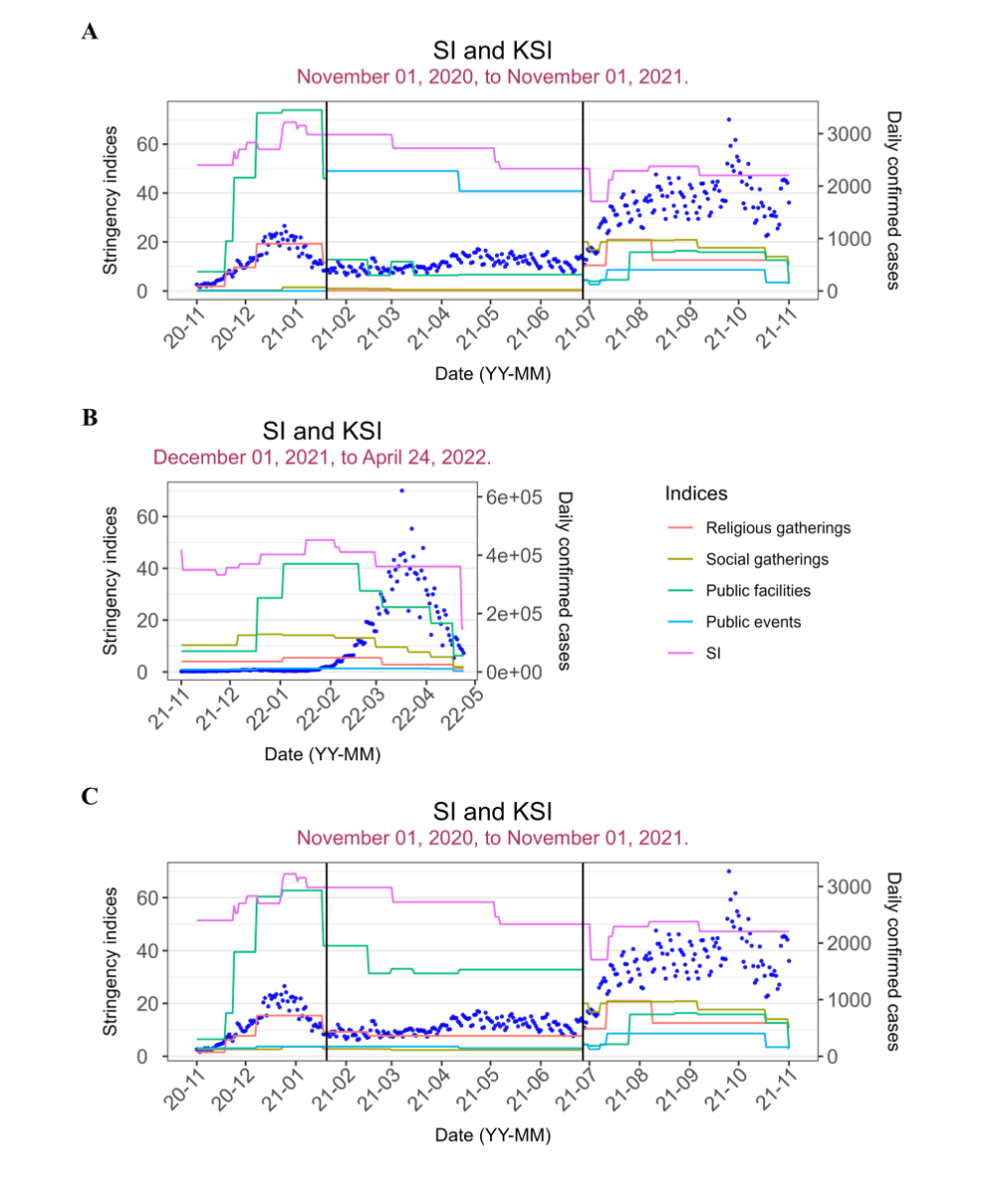
Variation of the KSIs and the OxCGRT SI with daily confirmed cases for (A) analysis periods 1, 2, and 4; (B) analysis period 5; and (C) analysis period 3+4. KSI: Korea Stringency Index; SI: Stringency Index.

Four KSIs for public events, religious gatherings, social gatherings, and public facilities were calculated using the feature importance values and score values of policies under each target policy and category, respectively. In addition to SI obtained from the OxCGRT data, their variation with daily confirmed cases for the 5 analysis periods is shown in Figure 3. SI had higher indices across all analysis periods. Public facilities had relatively higher stringency indices than the other KSIs from periods 1 to 5, followed by religious gatherings, social gatherings, and public events, respectively. Furthermore, we observed that the stringency indices of KSIs tended to increase with the number of daily confirmed cases and decrease with decreasing daily cases.

**Figure 4:**
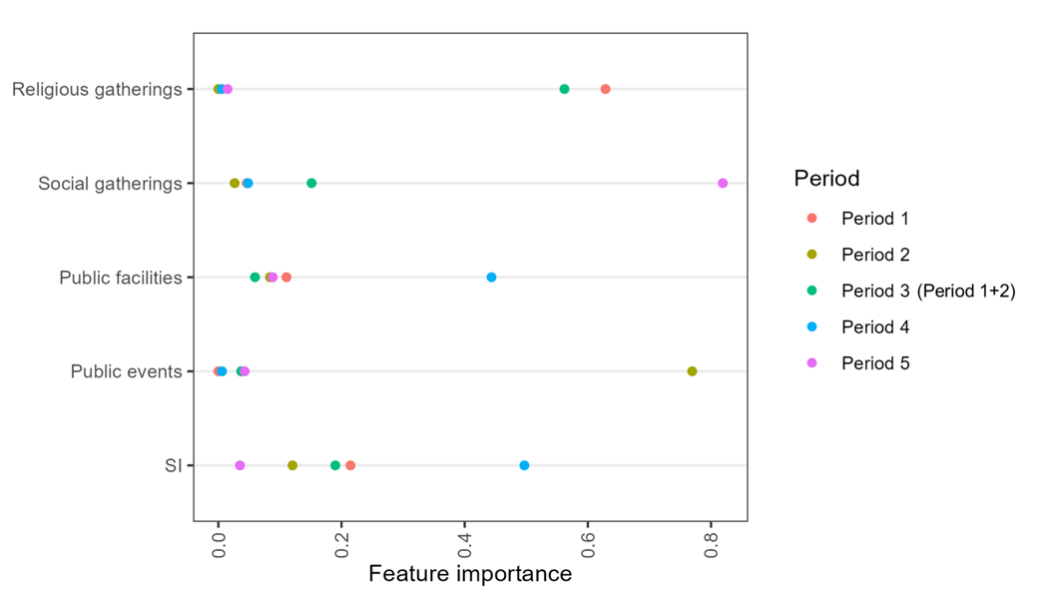
Feature importance for the Korea Stringency Indices and Oxford COVID-19 Government Response Tracker SI. SI: Stringency Index.

The KSIs had their feature importance below 0.20 in most analysis periods as shown in Figure 4. Exceptions were observed with public facilities in period 4, public events in period 2, social gatherings in period 5, and religious gatherings for period 1 and period 3.

### Correlation Analysis

**Figure 5:**
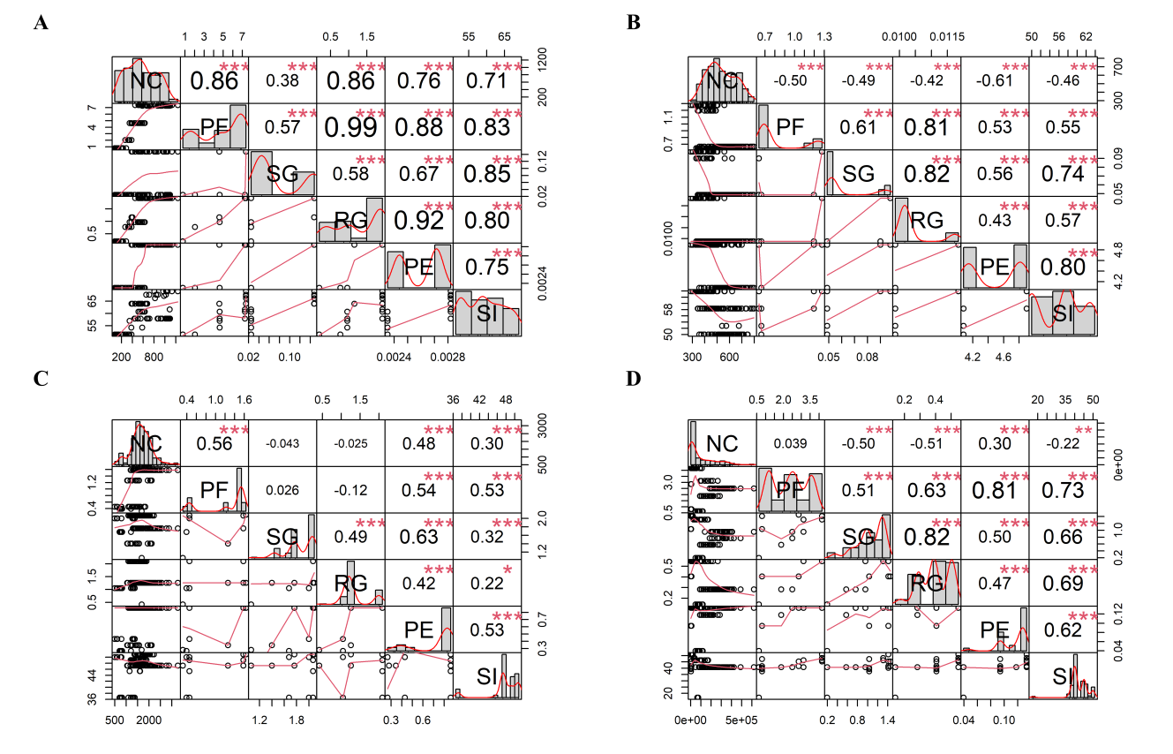
Correlation analysis plot for (A) analysis period 1; (B) analysis period 2; (C) analysis period 4; and (D) analysis period 5. The asterisk (*) indicates a statistically significant correlation. NC: new cases; PF: public facilities; PH: public events; RG: religious gatherings; SG: social gatherings; SI: Stringency Index.

The correlation analysis was carried out between KSIs, SI, and daily confirmed cases, as shown in Figure 5. In period 1 of the pandemic, all correlations were positive and statistically significant, with restrictions in the use of public facilities (*r*=0.86, *P*<.001), religious gatherings (*r*=0.86, *P*<.001), public events (*r*=0.76, *P*<.001), and SI (*r*=0.71, *P*<.001) being the strongest, respectively. Strong correlations were also observed between the KSIs, especially between public facilities and religious gatherings (*r*=0.99, *P*<.001), public facilities and public events (*r*=0.88, *P*<.001), and religious gatherings and public events (*r*=0.92, *P*<.001). However, a weak relationship was observed with restrictions in social gatherings (*r*=0.38, *P*<.001). In period 2 of the pandemic, a weaker and negative relationship between KSIs, SI, and daily confirmed cases was observed. However, there were strong correlations between public facilities and religious gatherings (*r*=0.81, *P*<.001) and religious and social gatherings (*r*=0.82, *P*<.001). Weak and sometimes negative correlations were observed in periods 4 and 5 between the KSIs and daily confirmed cases. However, strong correlations were observed between social and religious gatherings (*r*=0.82, *P*<.001) and public facilities and public events (*r*=0.81, *P*<.001) in period 5. The detailed results are summarized in Table S1 in Multimedia Appendix 1.

## Discussion

From our analysis, we observed that policies associated with operation hour restrictions and the number of gathering restrictions had a higher impact than other policies in the same analysis periods. The greatest impact was observed with the restrictions on the use of public facilities, especially in operating hour restrictions of cinemas, restaurants, coffee shops, indoor sports facilities, PC rooms, karaoke, and baths or saunas. The next impact was with policies restricting the number of people gatherings in public, religious, and social gatherings. For KSIs, their feature importance was below 0.20 in most analysis periods except public events in period 2, social gatherings in period 5, religious gatherings in periods 1 and 3, and public events in period 4. Although the stringency of SI was higher than KSIs, its feature importance with COVID-19 daily cases was lower than 0.40 across all analysis periods. Furthermore, we observed that different policies are effective at different analysis periods. Other periods of the pandemic require the enforcement of varying restriction policies. For example, the summer period is accompanied by a lot of indoor and outdoor activities and travel which increases the contact among people in the population, thus the rate of daily confirmed cases. To lower the wave of daily cases, the number of gathering restrictions and the use of public facilities, especially in PC rooms, restaurants and coffee shops, and cinemas would be more effective. These policies would reduce the mixing of people, allowing confirmed cases to be isolated and recovered. After flattening the curve, we can ease the strictness of using public facilities and social gatherings while maintaining a high stringency level on religious and public gatherings. Therefore, different policies have to be strengthened depending on the situation of the pandemic and significant activities happening in the country.

Correlation analysis showed a strong positive relationship between KSIs and daily confirmed cases in period 1 of the pandemic. A rise in the average daily confirmed cases corresponded with an increase in the stringency levels of the policies. The increase in the KSIs stringency indices leads to lower numbers of COVID-19 daily confirmed cases. In period 2, the relationship between the KSIs and daily confirmed cases was negative and weaker, maybe due to the easing of the strictness of the policies because of the flattening of the cases’ curve. From then on, the correlations stayed weak, and the relationship was either negative or positive, depending on the KSI. This can be attributed to easing the restriction policies’ strictness because most of the population was already vaccinated and opening the country back to normal operations. The correlation of SI was generally lower than those of the KSIs, showing that using KSIs to evaluate the effectiveness of restriction policies on COVID-19 cases is better than the OxCGRT SI. The KSIs provide more information about the effectiveness of the different policies at different target categories as the pandemic progresses.

Some argue that the COVID-19 pandemic caused by the contact-transmissible SARS-CoV-2 virus is our most significant health crisis [33]. However, its global impact on health, business, education, travel, international security, and other aspects of life cannot be disputed. Its outbreak exposed the weakness of health care systems, countries, and world preparedness in handling a sudden global crisis. Due to the lack of an effective treatment or vaccine, the pandemic elicited unprecedented restriction (or social distancing) policies to mitigate and suppress the spread of the virus. Around 110 countries have implemented at least 1 restriction policy against COVID-19 [26]. Standard policies included school closures, travel restrictions, bans on public gatherings, stay-at-home orders, closure of public transportation, emergency investments in the health care system, contact tracing, and investments in COVID-19 vaccines. Due to its high recovery rate of above 95% [34], the implementation of the restriction policies was also met with resistance from the general population as the social distancing period lengthened mainly toward vaccination and wearing masks [35-43]. Opposition came primarily from business holders, workers, and parents of school-going children because of the impact of these restrictions on education and businesses. However, evidence from several scientific studies and disease modeling continued to support the effectiveness of these policies on COVID-19 cases, thereby motivating governments to continue their implementation [44], especially with the emergence of more severe and transmissible SARS-CoV-2 variants like the delta and omicron variants, respectively [45-49].

The Korean government without resorting to any draconian “lockdown” policies like other countries of Uganda, China, the United States, etc [21,28,29], resorted to population testing combined with contact tracing, early isolation, free treatment of positive cases and digital technologies to fight the pandemic. Thirty-three restriction policies targeting public facilities, public events, social gatherings, and religious gatherings were implemented in Korea during the pandemic. These policies mainly targeted operating hour restrictions of public facilities or places and restrictions on the number of people gathering. Therefore, analysis of these policies to determine which government-implemented policies were most effective in managing the COVID-19 situation in Korea can provide a framework for responses that can be adopted against future pandemics. From our analysis, we observed the greatest impacts came from restrictions in public facilities, especially in operating hour restrictions of cinemas, restaurants, coffee shops, indoor sports facilities, PC rooms, karaoke, and baths or saunas, followed by restrictions to the number of people in public, religious and social gatherings. From the literature, an analysis of restriction policies in more than 90 countries showed the positive impact of containment policies like cancellation of public events, school and workplace closures, stay-at-home requirements, and restriction on gatherings, on the spread of COVID-19 [17]. Travel restrictions were shown to be effective in reducing the risk of imported SARS-CoV-2 cases [50]. Masking was found to be the most cost-effective nonpharmacological intervention implemented, delivering 4 times more impact than school closures and approximately double that of other mobility restrictions [51]. Gathering restrictions were the second most effective while international travel controls and public information campaigns had negligible effects [51]. From the above literature review, we consistently observe policies associated with gathering restrictions or restrictions on places where many people meet and mix like public places, workplaces, and schools, to always stand out among other policies, which is consistent with our findings of restrictions on public facilities and number of gathering restrictions.

The major limitation of our analysis was the collection of the restriction policy levels, which was done manually. This was time-consuming. Furthermore, since the policy levels were summarized from the KDCA website, some unnoticed changes may have been missed and thus not incorporated. In addition, the strictness levels and their compliance varied greatly between metropolitan areas like Seoul and Gyeonggi-do, which had a higher average number of daily cases, and the nonmetropolitan areas. However, we only focused on the national restriction levels when summarizing the restriction policy levels and calculated only nationwide-specific indices. Local or regional information was neglected and not incorporated when calculating indices or summarizing policy levels. Local or regional information must be considered as it is more insightful, especially considering countries such as the United States, which have large territories and large differences in regional policies. Furthermore, although our analysis focused only on Korea due to a lack of detailed information about the restriction policies of other countries, our method of calculating the stringency indices is simple and can easily be applied to other countries. However, the reliability of the developed indices will vary greatly depending on the types of policies implemented and the frequency of policy information provided by each country. In the future, the crawl-based semiautomated systems can be adapted to collect policy data, after which the validity of the data can be confirmed manually. To increase the generalizability of the result, we can consider developing the region-specific stringency indices using regional data. Furthermore, considering the population demographic information like age may provide more in-depth results and compliance levels across age groups. In addition, more robust methods of calculating indices need to be explored.

In conclusion, nonpharmacological restriction policies that aim for physical distancing have a strong potential to suppress the spread of COVID-19 and lead to a smaller number of overall cases. From our analysis, we observed that restrictions on the use of public facilities focusing on restaurants, coffee shops, cinemas, PC rooms, saunas or baths, indoor sports facilities, and restrictions on the number of gatherings, have played a significant role in slowing down the spread of the disease, thus buying time for the health care systems and governments. Given the high impact of restriction policies on public facilities, a nonpharmacological restriction policy framework can be designed with restrictions on public facilities being the main focus for diseases that spread through contact between people in the population. However, different periods call for enforcing different policies as their effectiveness can vary during the pandemic. Since nonpharmacological restriction policies alone cannot effectively combat a spreading pandemic, other factors like an effective health care plan or treatment, population demographics, and population mobility must be considered for either long-term or short-term impact. The compliance from the public must also be considered for both short and long-term effectiveness.

## Appendix

Multimedia Appendix 1 Detailed results of the correlation analysis.

## Abbreviations

KDCA: Korea Disease Control and Prevention Agency
KSI: Korea Stringency Index
OWID: Our World in Data
OxCGRT: Oxford COVID-19 Government Response Tracker
RF: random forest
SI: Stringency Index

## Data Availability

The data sets generated or analyzed during this study are available from the corresponding author on reasonable request.
