# Supplementary material for: Development of New Stringency Indices for Nonpharmacological Social Distancing Policies Implemented in Korea During the COVID-19 Pandemic: Random Forest Approach
Source: JMIR Public Health Surveill. 2024 Jan 8;10:e47099. doi: 10.2196/47099 (PMC10775907; doi:10.2196/47099)
Supplement: Multimedia Appendix 1 [file publichealth_v10i1e47099_app1.docx]

**Table S1.** Detailed results of the correlation analysis. Values outside the parenthesis are Pearson correlation coefficients, and the values inside the parenthesis are *P* values.

| Variable 1 | Variable 2 | Period 1 | Period 2 | Period 4 | Period 5 |
| --- | --- | --- | --- | --- | --- |
| New cases | Public facilities | 0.864 (*P*<.001) | -0.498 (*P*<.001) | 0.563 (*P*<.001) | 0.039 (.606) |
| New cases | Social gatherings | 0.382 (*P*<.001) | -0.493 (*P*<.001) | -0.043 (.633) | -0.497 (*P*<.001) |
| New cases | Religious gatherings | 0.865 (*P*<.001) | -0.422 (*P*<.001) | -0.025 (.777) | -0.506 (*P*<.001) |
| New cases | Public events | 0.758 (*P*<.001) | -0.608 (*P*<.001) | 0.479 (*P*<.001) | 0.297 (*P*<.001) |
| New cases | SI^a^ | 0.715 (*P*<.001) | -0.460 (*P*<.001) | 0.300 (*P*<.001) | -0.223 (.003) |
| Public facilities | Social gatherings | 0.566 (*P*<.001) | 0.612 (*P*<.001) | 0.026 (.768) | 0.513 (*P*<.001) |
| Public facilities | Religious gatherings | 0.987 (*P*<.001) | 0.810 (*P*<.001) | -0.119 (.183) | 0.634 (*P*<.001) |
| Public facilities | Public events | 0.875 (*P*<.001) | 0.529 (*P*<.001) | 0.540 (*P*<.001) | 0.811 (*P*<.001) |
| Public facilities | SI | 0.828 (*P*<.001) | 0.545 (*P*<.001) | 0.533 (*P*<.001) | 0.734 (*P*<.001) |
| Social gatherings | Religious gatherings | 0.580 (*P*<.001) | 0.818 (*P*<.001) | 0.492 (*P*<.001) | 0.819 (*P*<.001) |
| Social gatherings | Public events | 0.667 (*P*<.001) | 0.560 (*P*<.001) | 0.631 (*P*<.001) | 0.496 (*P*<.001) |
| Social gatherings | SI | 0.845 (*P*<.001) | 0.742 (*P*<.001) | 0.319 (*P*<.001) | 0.662 (*P*<.001) |
| Religious gatherings | Public events | 0.917 (*P*<.001) | 0.428 (*P*<.001) | 0.419 (*P*<.001) | 0.469 (*P*<.001) |
| Religious gatherings | SI | 0.800 (*P*<.001) | 0.568 (*P*<.001) | 0.217 (.014) | 0.688 (*P*<.001) |
| Public events | SI | 0.754 (*P*<.001) | 0.796 (*P*<.001) | 0.530 (*P*<.001) | 0.616 (*P*<.001) |

^a^SI: Stringency Index.
